# Mortality during tuberculosis treatment in South Africa using an 8-year analysis of the national tuberculosis treatment register

**DOI:** 10.1038/s41598-021-95331-w

**Published:** 2021-08-05

**Authors:** Muhammad Osman, Cari van Schalkwyk, Pren Naidoo, James A. Seddon, Rory Dunbar, Sicelo S. Dlamini, Alex Welte, Anneke C. Hesseling, Mareli M. Claassens

**Affiliations:** 1 Desmond Tutu TB Centre, Department of Paediatrics and Child Health, Faculty of Medicine and Health Sciences, Stellenbosch University, Francie van Zijl Drive, Parow, Cape Town, 7505 South Africa; 2 DSI-NRF South African Centre of Excellence in Epidemiological Modelling and Analysis (SACEMA), Stellenbosch University, Stellenbosch, South Africa; 3 Department of Infectious Diseases, Imperial College London, London, UK; 4 Research Information Monitoring, Evaluation & Surveillance (RIMES), National TB Control & Management Cluster, National Department of Health, Pretoria, South Africa; 5 Department of Biochemistry and Microbiology, School of Medicine, University of Namibia, Bach Street, Windhoek, Namibia

**Keywords:** Health services, Risk factors, HIV infections, Tuberculosis, Public health, Epidemiology

## Abstract

In 2011, the South African HIV treatment eligibility criteria were expanded to allow all tuberculosis (TB) patients lifelong ART. The impact of this change on TB mortality in South Africa is not known. We evaluated mortality in all adults (≥ 15 years old) treated for drug-susceptible TB in South Africa between 2009 and 2016. Using a Cox regression model, we quantified risk factors for mortality during TB treatment and present standardised mortality ratios (SMR) stratified by year, age, sex, and HIV status. During the study period, 8.6% (219,618/2,551,058) of adults on TB treatment died. Older age, male sex, previous TB treatment and HIV infection (with or without the use of ART) were associated with increased hazard of mortality. There was a 19% reduction in hazard of mortality amongst all TB patients between 2009 and 2016 (adjusted hazard ratio: 0.81 95%CI 0.80–0.83). The highest SMR was in 15–24-year-old women, more than double that of men (42.3 in 2016). Between 2009 and 2016, the SMR for HIV-positive TB patients increased, from 9.0 to 19.6 in women, and 7.0 to 10.6 in men. In South Africa, case fatality during TB treatment is decreasing and further interventions to address specific risk factors for TB mortality are required. Young women (15–24-year-olds) with TB experience a disproportionate burden of mortality and interventions targeting this age-group are needed.

## Introduction

In 2018, an estimated 10 million people developed tuberculosis (TB) disease worldwide and, among adults (≥ 15 years old), 64% of cases occurred in men^1^. Despite progress towards reducing TB mortality, it remained the leading cause of death from an infectious disease globally, with an estimated 1.5 million (15%) deaths^1^. A systematic review found that case fatality during TB treatment ranged from 1.8 to 33.3%; male sex was identified as a risk factor for death^2^. Understanding mortality in adults on TB treatment presents an opportunity to plan interventions to reduce mortality amongst individuals already engaged with health services.

In sub-Saharan Africa, the dual HIV and TB epidemics have been well described. Prior to the rollout of antiretroviral therapy (ART) the observed rise in TB case fatality ratio (CFR) was linked to increasing HIV prevalence^3^. In South Africa, routine HIV testing was implemented in 2010^4^ and in 2012 the ART policy expanded so that all HIV-positive TB patients were eligible for lifelong ART^5^. ART uptake in South Africa has been variable with adult women showing the greatest uptake of ART^6^.

The dramatic effect of ART on reducing mortality among HIV-positive TB patients has been documented under both trial^7^ and routine programmatic conditions^8^. In low- and middle-income countries, the effect of wide-scale ART rollout on mortality has varied by sex, with greater reductions in mortality observed in women^9–11^. A recent systematic review showed a mortality reduction of 44–71% among HIV-positive TB patients with the use of ART^8^. In South Africa, modelling work has shown that while the ART rollout has contributed to a substantial number of lives saved, almost 2.2 million people died between 2000 and 2014 due to delays in the rollout^12^. Understanding the changes in mortality during TB treatment by age and sex, in the context of the scale up of ART, may provide insights into how health programs can deliver targeted services to further reduce TB mortality in South Africa and globally.

The impact of the revised TB, HIV and ART policies on mortality during TB treatment in South Africa, and how the impact has differed by sex, is not well known. Using an individual patient-based national electronic TB treatment register of drug-susceptible TB treatment we documented the changes in TB mortality compared to the general population mortality and identified risk factors for death during TB treatment.

## Methods

### Context

South Africa had an estimated population of 55.7 million in 2016, a 11.6% increase from 49.9 million in 2009^12^. The country offers a district health service with 52 health districts delivering public health services, across 9 provinces. In 2018, South Africa had an estimated TB incidence of 520/100,000 per year and approximately 59% of TB patients were HIV-positive^1^. Laboratory diagnosis of TB in South Africa utilised smear microscopy, culture and line probe assay until 2011, after which the GeneXpert MTB/RIF assay (Xpert; Cepheid, Sunnyvale, CA) was progressively introduced for the testing of all patients with presumptive TB.

### Electronic TB register (ETR.Net)

In South Africa, all patients were presumed to have drug-susceptible TB, unless drug susceptibility testing demonstrated resistance, and were routinely registered in a paper-based register at a TB treatment facility when initiating treatment. This register was validated by facility managers and sub-district co-ordinators before it was captured into an electronic TB register (ETR.Net), and aggregated at district, provincial and national level^13^. We obtained national and provincial ETR.Net dispatch files and undertook a process of merging, de-duplication, and data cleaning. ETR.Net includes a unique identifier and this was used to identify duplicate records that were merged before deletion to provide complete data on HIV, treatment dates and outcomes. HIV data in ETR.Net is variably completed and may include HIV status, CD4 count, and the use of cotrimoxazole preventive therapy or ART. Within ETR.Net each patient was recorded for the first time as a newly registered patient and could then move or be transferred between facilities in the country. A cohort including all newly registered TB patients was used to report on TB case finding and a reporting cohort accounting for patient movement during treatment was used for the reporting of TB treatment outcomes. For this analysis, the South African reporting cohort was used.

### The Thembisa model

Estimates of population denominators, HIV prevalence, and mortality were extracted from the Thembisa project (https://www.thembisa.org/), a publicly available mathematical model of HIV and general demographic statistics^14^. The model uses age and sex-specific mortality estimates based on an analysis of South African cause-of-death statistics and the South African National Burden of Disease study, and projects mortality from 2016 to 2030^12^.

### Design

This study is a retrospective cohort analysis of mortality among all adults (≥ 15 years) in South Africa routinely started on drug-susceptible TB treatment between 1 January 2009 and 31 December 2016.

### Definitions

Consistent with global reporting of TB we considered adults as those ≥ 15 years old^1,15^. Table 1 includes the definitions used for variables recorded within ETR.Net^15^. For outcome definitions the standard WHO and national TB program definitions were used^15,16^. CFR was calculated as the proportion of deaths as compared to the total number of people on TB treatment. The total number of people on TB treatment included patients with unknown outcomes. Two additional outcome variables were constructed to evaluate unfavourable outcomes (‘Outcome 2’) and mortality among patients with known final outcomes (‘Outcome 3’). Person-time was calculated as the difference between the TB treatment start date and treatment outcome date, representing the person-years on TB treatment. TB deaths refer to deaths due to any cause during the TB treatment episode and underlying cause of death was not evaluated. This is consistent with the national TB program and WHO definition^15,16^.

**Table 1:** Definitions used in the study based on variables recorded within ETR.Net and classified according to the WHO reporting framework.

Variable	Categories	Definition
Previous TB treatment history	Newly treated	Patients who had no reported previous TB treatment or had received less than 4 weeks of TB treatment at any stage
	Retreatment	Patients who received more than 4 weeks of anti-tuberculosis treatment previously, regardless of the time since the previous episode
Site of disease	Pulmonary TB	Site of disease included any pulmonary involvement
	Extrapulmonary TB	Site of disease exclusively affecting any organ other than the lung parenchyma
Extent of disease^a^	Disseminated	ICD10 codes for TB meningitis or miliary TB were combined
	Not disseminated	All other ICD10 codes
HIV status^b^	HIV-positive	Determined using HIV test results, CD4 results, documentation of co-trimoxazole preventive therapy or use of ART. ART use does not include date of starting ART and cannot differentiate ART before TB or those started on ART during TB treatment
	HIV-negative	Documented negative results in TB treatment register
	HIV unknown	HIV status unable to be determined from results or inference
Outcome 1^c^	Success	Cured or treatment completed
	Died	Death due to any cause during the TB treatment episode
	Lost-to-follow-up	Patients who interrupted TB treatment for 2 consecutive months or patients where a treatment outcome is not allocated and an outcome of ‘not evaluated’ was recorded in ETR.Net
	Failed	Patients who were microbiologically positive at the start of treatment and remain or become positive after at least 5 months of TB treatment
	Drug resistance	Patient diagnosed with drug resistance after 12 weeks of TB treatment
	Transferred out	Patient who was referred to a facility in another district and the final TB treatment outcome is not known
Outcome 2	Favourable	Patients successfully treated for TB (cured or completed)
	Unfavourable	A combined definition of all negative outcomes including died, lost-to-follow-up, failed, drug resistance, or transferred out
Outcome 3	Dead	Death due to any cause during the TB treatment episode
	Alive	Patients who were known not to have died at the end of TB treatment including success, failed or drug resistance. This excludes patients who were lost-to-follow-up or transferred out where mortality is not known

ART: antiretroviral therapy; ETR.Net: Electronic tuberculosis register; ICD10: International classification of disease 10; TB: tuberculosis.

^a^Extent of disease could only be ascertained where an ICD10 code was documented.

^b^HIV status is typically recorded at the start of TB treatment but may be updated during TB treatment.

^c^Outcomes are assigned by routine treating clinicians and ETR.Net includes pre-programmed algorithms to verify outcomes consistent with WHO and National Department of Health definitions.

### Statistical analysis

We conducted descriptive statistics for categorical variables and stratified these by HIV status. Changes over time were evaluated by year and graphically illustrated. CFRs were calculated by age, sex and HIV-status and provide a conservative estimate of mortality as the true mortality status of TB patients who were transferred out, lost to follow up or not evaluated at the end of treatment is unknown. A Cox proportional hazards model was used to determine the unadjusted and adjusted hazard ratios (HRs) with 95% confidence intervals (CI) for predictors of death. Following univariate analyses, predictors were added incrementally observing the change in significance of the likelihood ratio test of each model and collinearity between variables was considered before developing the final adjusted model. The Thembisa model provided the expected mortality rate per demographic category for each year for the general population of South Africa^14^. The expected mortality is informed by all causes and includes TB. The product of the expected mortality and person-years in the TB reporting cohort provided the number of expected deaths in the TB cohort. The standardised mortality ratios (SMR) were calculated as the ratio between the observed TB deaths and the expected deaths within the TB cohort. The data analysis for this paper was generated using SAS software, Version 9.4 of the SAS System for Windows. Copyright 2002–2012 SAS Institute Inc. SAS and all other SAS Institute Inc. product or service names are registered trademarks or trademarks of SAS Institute Inc., Cary, NC, USA.

### Ethics

The National Department of Health, National TB program provided permission for this analysis and approval was received from the Stellenbosch University Health Research Ethics Committee (N16/07/088). A waiver of informed consent was granted from Stellenbosch University Health Research Ethics Committee. All methods were carried out in accordance with relevant guidelines and regulations.

## Results

The South African reporting cohort for drug-susceptible TB included 2,551,058 adults, 55.9% men, treated between 2009 and 2016 (Table 2). HIV testing and recording increased from 54.8% of individuals with TB having a known HIV status in 2009, to 96.7% in 2016 (Table 2 and Supplementary Figure 1).

**Table 2:** Demographic and clinical characteristics of all adults (≥ 15 years) routinely treated for drug-susceptible TB in the South African reporting cohort, stratified by HIV status, aggregated for 2009–2016.

		HIV unknown		HIV-positive		HIV-negative		Total	
		n	col %	n	col %	n	col %	n	col %
Total		395,486	100	1,443,557	100	712,015	100	2,551,058	100
Age category	15–24 years	59,020	14.9	126,747	8.8	157,973	22.2	343,740	13.5
	25–34 years	116,147	29.4	517,793	35.9	157,445	22.1	791,385	31.0
	35–44 years	99,473	25.2	480,515	33.3	120,213	16.9	700,201	27.4
	45–54 years	64,703	16.4	229,060	15.9	121,999	17.1	415,762	16.3
	55–64 years	33,439	8.5	73,147	5.1	87,547	12.3	194,133	7.6
	65 + years	22,704	5.7	16,295	1.1	66,838	9.4	105,837	4.1
Sex	Female	162,900	41.2	703,339	48.7	257,618	36.2	1,123,857	44.1
	Male	232,586	58.8	740,218	51.3	454,397	63.8	1,427,201	55.9
Previous TB disease	New	334,507	84.6	1,240,553	85.9	613,707	86.2	2,188,767	85.8
	Retreatment	60,979	15.4	203,004	14.1	98,308	13.8	362,291	14.2
Site of disease	EPTB	50,802	12.8	211,941	14.7	60,916	8.6	323,659	12.7
	PTB	344,684	87.2	1,231,616	85.3	651,099	91.4	2,227,399	87.3
Disseminated TB^a^	Not disseminated	350,036	97.0	1,283,236	95.7	656,757	98.6	2,290,029	96.7
	Disseminated	10,645	3.0	57,059	4.3	9495	1.4	77,199	3.3
Outcome 1^b^	Cured/completed	266,336	67.3	1,065,118	73.8	566,009	79.5	1,897,463	74.4
	Died	42,478	10.7	138,992	9.6	38,577	5.4	220,047	8.6
	Drug resistance	2310	0.6	9297	0.6	3881	0.5	15,488	0.6
	Failed	1426	0.4	4117	0.3	3403	0.5	8946	0.4
	Loss to follow up	57,240	14.5	157,657	10.9	75,641	10.6	290,538	11.4
	Transferred out	25,696	6.5	68,376	4.7	24,504	3.4	118,576	4.6
Outcome 2^c^	Favourable	266,336	67.3	1,065,118	73.8	566,009	79.5	1,897,463	74.4
	Unfavourable	129,150	32.7	378,439	26.2	146,006	20.5	653,595	25.6
Outcome 3^d^	Alive	270,072	86.4	1,078,532	88.6	573,293	93.7	1,921 897	89.7
	Dead	42,478	13.6	138,992	11.4	38,577	6.3	220,047	10.3
Year of TB treatment	2009	161,905	40.9	140,465	9.7	59,007	8.3	361,377	14.2
	2010	93,587	23.7	188,840	13.1	78,089	11	360,516	14.1
	2011	51,202	12.9	209,485	14.5	93,095	13.1	353,782	13.9
	2012	33,845	8.6	196,722	13.6	92,669	13	323,236	12.7
	2013	22,869	5.8	188,816	13.1	98,050	13.8	309,735	12.1
	2014	14,563	3.7	192,718	13.4	103,816	14.6	311,097	12.2
	2015	9413	2.4	173,058	12	98,118	13.8	280,589	11.0
	2016	8102	2	153,453	10.6	89,171	12.5	250,726	9.8

ETR.Net: electronic tuberculosis register; ICD10: International classification of disease 10; TB: tuberculosis; EPTB: extrapulmonary TB.

^a^Disseminated TB was defined using the ICD10 coding, with miliary TB and neurological TB classified as disseminated TB and all other ICD10 codes as not disseminated. Missing ICD10 codes n = 183,830.

^b^Outcome 1, as classified in ETR.Net, combining cure and complete for success; and all patients without outcomes as lost to follow up.

^c^Outcome 2, a binary outcome with success as the only favourable outcome and all other outcomes considered unfavourable.

^d^Outcome 3, patients in whom a final vitality status is unknown (lost to follow up, moved or not evaluated) n = 409,114 were excluded.

### Case fatality ratios

**Figure 1:**
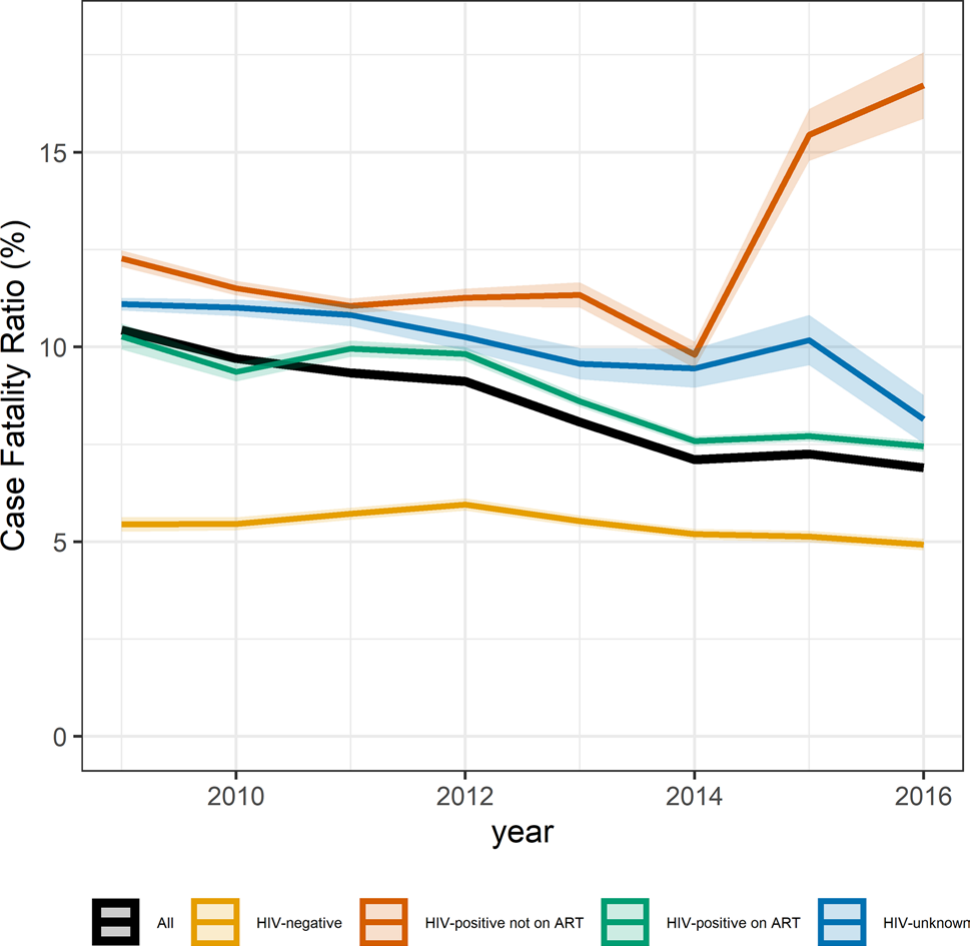
Overall South African case fatality ratio and 95%CI for adults (≥ 15 years) routinely treated for drug-susceptible tuberculosis, stratified by HIV status 2009–2016. ART: antiretroviral therapy; TB: tuberculosis. Case fatality ratio is calculated as percentage of deaths among all those with TB, regardless of known or unknown outcomes.

**Figure 2:**
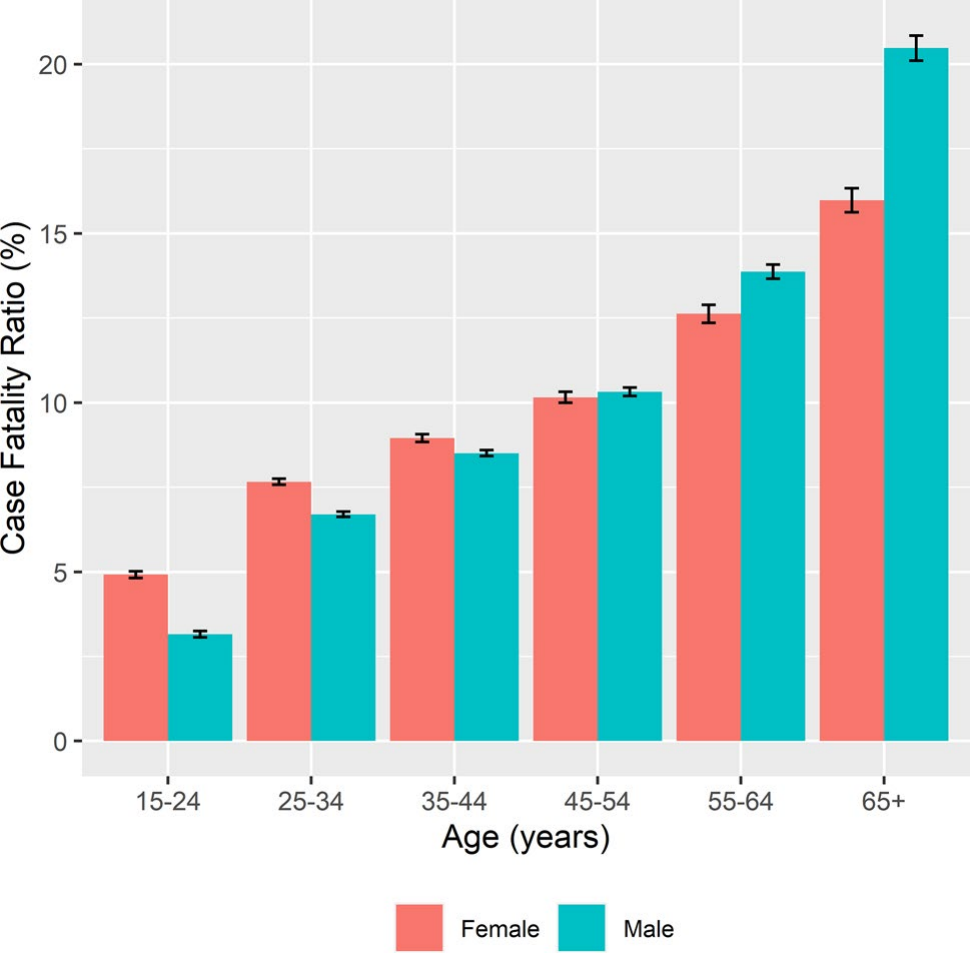
Case fatality ratio of adults (≥ 15 years) on drug-susceptible tuberculosis treatment by age category and sex for the combined period 2009–2016, South Africa. Case fatality ratio is calculated as percentage of deaths among all those with TB, regardless of known or unknown outcomes.

Over the 8 years 219,618 deaths were documented. The TB CFR decreased from 10.4% in 2009, to 6.9% in 2016 (Fig. 1). The CFR for HIV-negative individuals increased from 5.5% in 2009 to 6.0% in 2012 and then decreased to 4.9% in 2016. The CFR for HIV-positive patients not on ART increased over the study period to 16.1% in 2016. The CFR for HIV-positive individuals on ART decreased from 10.2% in 2009 to 7.4% in 2016 (Fig. 1). The CFR differed by age and sex: among 15–24-year-olds, 4.9% of women died compared to 3.2% of men. Among 65+ year-olds, men had a CFR of 20.5% compared to 16.0% in women over the entire period (Fig. 2). Treatment success increased from 70.2% in 2009 to 80.4% in 2016. Lost-to-follow-up decreased from 12.4% in 2009 to 9.4% in 2016. Unfavourable outcomes decreased from 29.8% in 2009 to 19.7% in 2016. Outcome 3, death among adults with known TB treatment outcomes, decreased from 12.8% in 2009 to 7.9% in 2016 (Supplementary Table 1).

### Risk factors for death

Increasing age was associated with increased hazard of death with 65+ year-olds having an adjusted hazard ratio (aHR) of 5.22 (95%CI 5.10–5.33) compared to 15–24-year-olds. Compared to HIV-negative TB patients, HIV-positive patients not on ART had an aHR of 2.71 (95%CI 2.67–2.75); patients with unknown HIV status had an aHR 2.19 (95%CI 2.15–2.22) and HIV-positive on ART had an aHR of 1.84 (95%CI 1.81–1.86). Previous TB treatment history and extrapulmonary TB had an increased hazard of death with the risk declining over time (Table 3). We explored effect modification by sex and time in stratified models and found differences in point estimates for age categories and HIV status (Supplementary Tables 2 and 3).

**Table 3:** Univariate and multivariable Cox regression model predicting hazard and adjusted hazard ratio of death among adults (≥ 15 years) with drug-susceptible TB and known vitality status, South Africa, 2009–2016 (n = 2,122,419).

		Total	Dead	Outcome 3^c^	Univariate Cox regression^d^	Multivariable Cox regression^d^
		n	n	%	HR (95%CI)	aHR (95%CI)
Age category	15–24 years	279,452	13,922	5.0	Reference	Reference
	25–34 years	644,837	55,122	8.5	1.73 (1.7–1.76)	1.42 (1.4–1.45)
	35–44 years	584,348	59,160	10.1	2.06 (2.02–2.09)	1.66 (1.63–1.69)
	45–54 years	354,032	41,649	11.8	2.41 (2.37–2.46)	2.11 (2.07–2.15)
	55–64 years	167,905	25,480	15.2	3.19 (3.13–3.26)	3.15 (3.09–3.22)
	65+ years	91,845	19,053	20.7	4.54 (4.44–4.64)	5.22 (5.1–5.33)
Sex	Male	1,172,409	121,491	10.4	Reference	Reference
	Female	950,010	92,895	9.8	0.94 (0.93–0.95)	0.95 (0.94–0.95)
HIV status	HIV-negative	607,943	37,971	6.2	Reference	Reference
	HIV unknown	308,414	40,629	13.2	2.14 (2.11–2.17)	2.17 (2.13–2.2)
	HIV-positive not on ART	419,369	59,755	14.2	2.32 (2.3–2.35)	2.66 (2.62–2.69)
	HIV-positive on ART	786,693	76,031	9.7	1.55 (1.53–1.57)	1.86 (1.83–1.88)
Previous TB treatment	New	1,839,987	176,943	9.6	Reference	Reference
	Retreatment	282,432	37,443	13.3	1.3 (1.29–1.32)	1.27 (1.26–1.29)
Site of disease	PTB	1,861,447	177,375	9.5	Reference	reference
	EPTB	260,972	37,011	14.2	1.43 (1.42–1.45)	1.41 (1.39–1.42)
Disseminated TB^a,b^	Not disseminated	1,913,826	183,896	9.6	Reference	
	Disseminated	62,196	12,556	20.2	2.05 (2.02–2.09)	
Year of TB treatment	2009	291,600	36,594	12.5	Reference	Reference
	2010	285,348	33,838	11.9	0.94 (0.93–0.96)	0.97 (0.96–0.99)
	2011	292,643	32,185	11.0	0.87 (0.86–0.88)	0.93 (0.92–0.95)
	2012	271,845	28,754	10.6	0.84 (0.83–0.85)	0.96 (0.94–0.97)
	2013	262,554	24,402	9.3	0.75 (0.74–0.76)	0.92 (0.91–0.94)
	2014	251,812	21,527	8.5	0.7 (0.69–0.71)	0.89 (0.88–0.91)
	2015	246,557	20,018	8.1	0.66 (0.64–0.67)	0.85 (0.84–0.87)
	2016	220,060	17,068	7.8	0.63 (0.61–0.64)	0.81 (0.8–0.83)

ART: antiretroviral therapy; EPTB: extrapulmonary TB; ICD10: International classification of disease 10; PTB: pulmonary TB; TB: tuberculosis.

p-values not shown, all p-values in multi variable model significant < 0.0001.

^a^Disseminated TB not added to final model due to collinearity with site of TB disease.

^b^Disseminated TB included 146,397 with unknown classification due to missing ICD10 codes.

^c^Outcome 3: calculated as percentage of deaths among those with known outcomes where person time on treatment was documented.

^d^Cox regression models restricted to individuals with known final vitality status. Unknown outcomes (lost to follow up, moved or not evaluated) n = 409,114 excluded.

### Standardised mortality ratios

**Figure 3:**
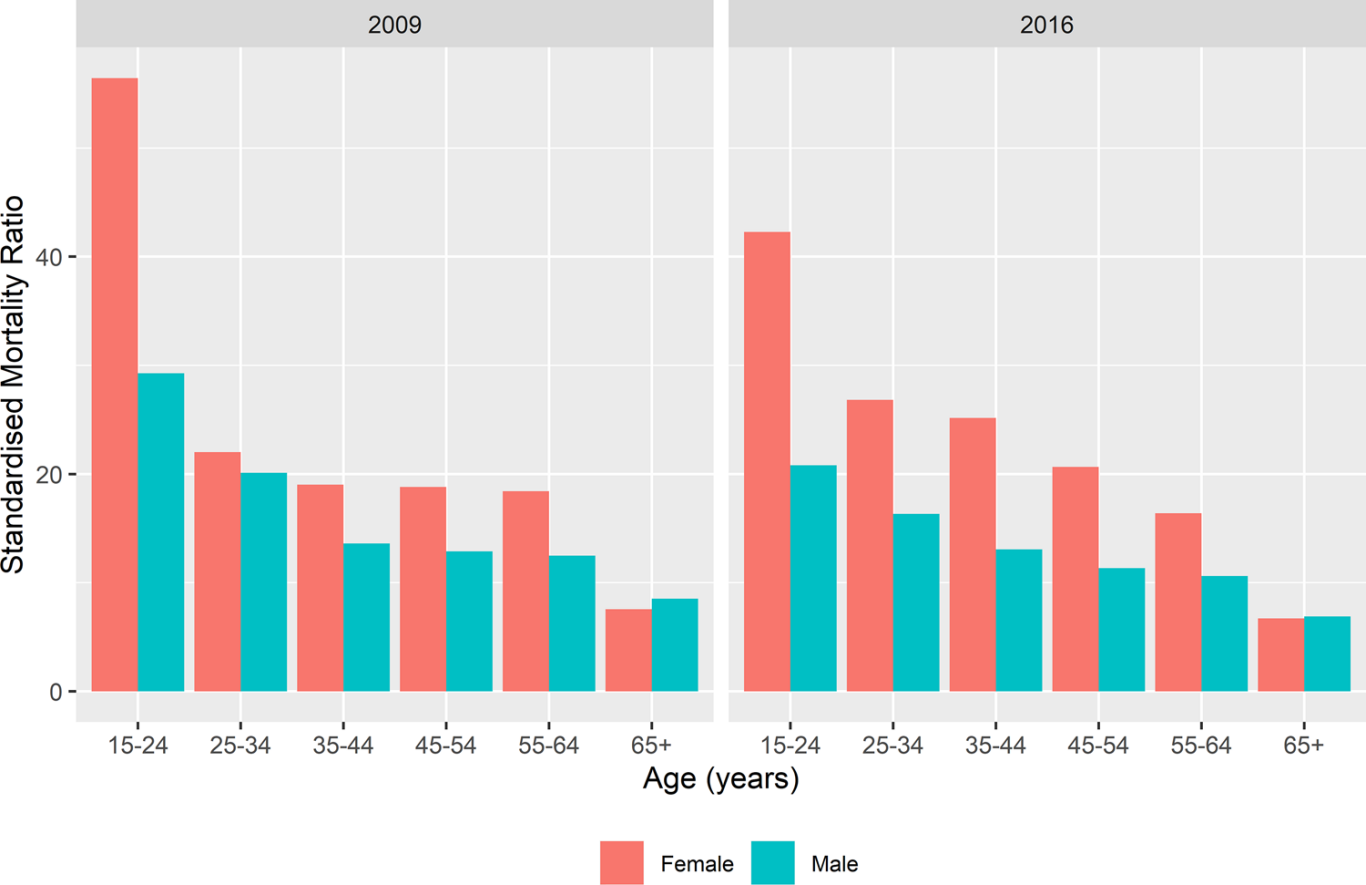
Standardised mortality ratio of adults (≥ 15 years) on drug-susceptible tuberculosis treatment by age category and sex for 2009 and 2016, South Africa.

**Figure 4:**
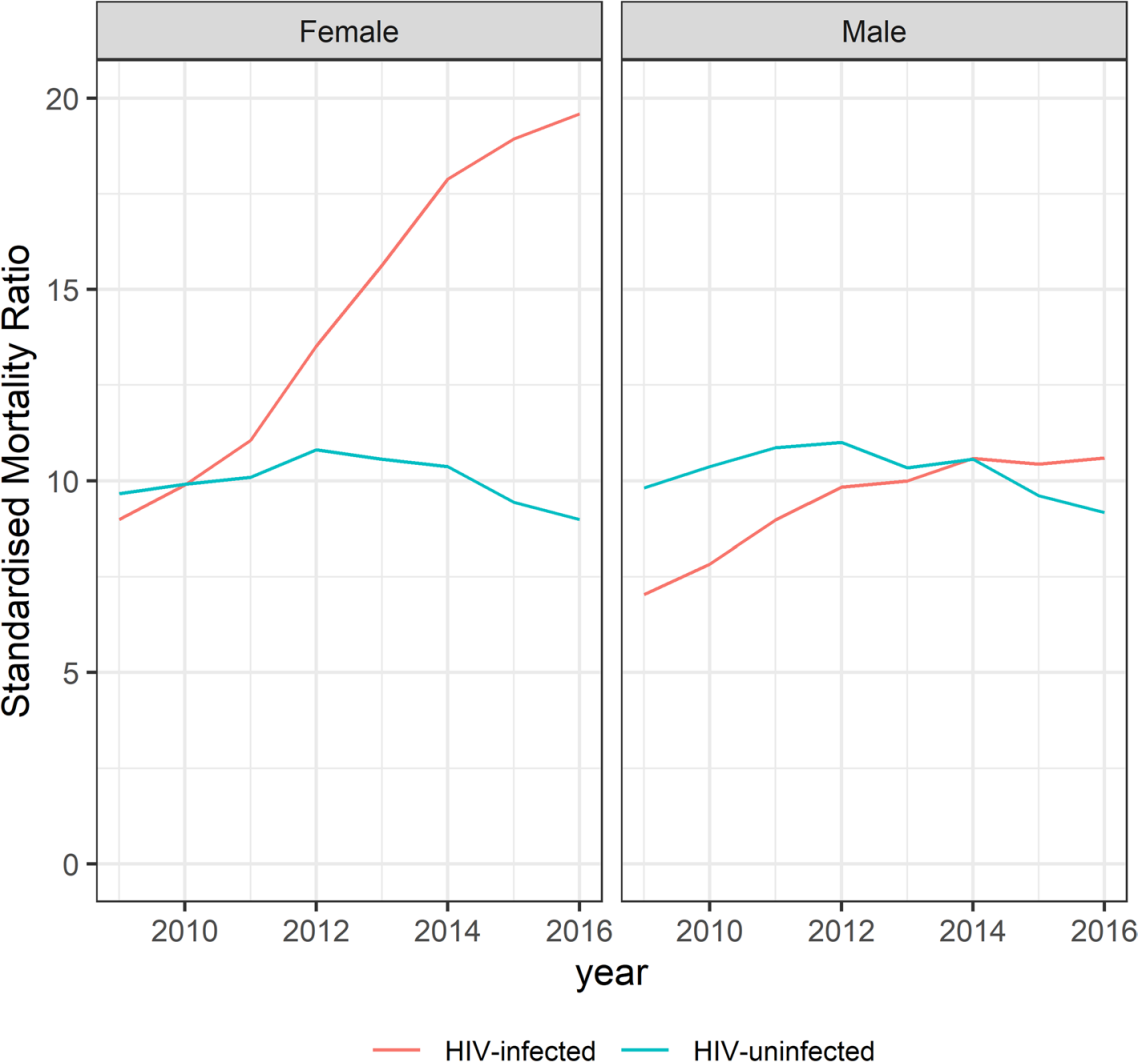
Standardised mortality ratios by HIV and sex of adults (≥ 15 years) on drug-susceptible tuberculosis treatment in South Africa, 2009–2016.

SMRs for age, sex and HIV were calculated over time. Women, 15–24 years old with TB had a SMR of 56.4 in 2009 compared to 42.3 in 2016. The SMR for men of the same age was 29.3 in 2009 and 20.8 in 2016. The SMR decreased by age and for 65+ year-olds the SMR for men was 6.9 and for women was 6.7 in 2016 (Fig. 3 and Supplementary Figure 2). Among HIV-positive TB patients the SMRs increased over time, for women the SMR increased from 9.0 in 2009 to 19.6 in 2016 and for men from 7.0 in 2009 to 10.6 in 2016. For HIV-negative TB patients, there was limited change in the SMR over time (Fig. 4).

## Discussion

TB CFR during treatment in South Africa is declining but remains high, with 6.9% of adult TB patients dying in 2016. Older age, male sex, previous TB treatment history and HIV status (with or without the use of ART) were associated with increased hazard of death during TB treatment. When compared to mortality in the general population, younger patients with TB had a substantially greater SMR, the highest in young women (15–24-year-olds). The SMR for HIV-positive TB patients increased over time, with a sharper increase among HIV-positive women compared to men.

South Africa has seen an overall decrease in the TB CFR, but stratification by HIV status and ART is crucial to understand underlying patterns. While an overall decline in the CFR to 6.9% in 2016 was documented, when disaggregated by HIV status, the CFR clearly showed an increase in HIV-positive TB patients not on ART, very little change for HIV-negative individuals, and the steepest decline in TB CFR in HIV-positive TB patients on ART. By 2016, more than 94% of HIV-positive TB patients were on ART and the increasing CFR among HIV-positive patients not on ART therefore reflected the small minority of HIV-positive patients. This group likely reflects those most disengaged with the health system and are a priority group for intervention and ART initiation. During this period, South Africa expanded its HIV testing program, scaled up ART for HIV-positive patients^17^, and in 2011, introduced Xpert as routine testing for presumptive TB patients^18^. Prior to these efforts more people would have experienced diagnostic delays and death prior to TB diagnosis or treatment initiation resulting in higher levels of under-reporting of total TB mortality. In addition, as drug susceptibility testing for all presumptive TB patients was not previously available, CFRs in earlier years may have also been higher due to those with undiagnosed drug-resistant TB. It is probable that the TB treatment category ‘retreatment’ included more drug-resistant TB in the earlier years of the cohort. This is supported by the stratified analyses which showed the aHR for retreatment decreased from aHR = 1.33 (95%CI 1.3–1.37) in 2009 to aHR = 1.09 (95%CI 1.04–1.15) in 2016 (Supplementary Table 3).

We documented an increased hazard of death with increasing age and among men and this has been described with older age in previous studies^2^. The increased hazard of death among 65+ year-olds compared to 15–24-year-olds may relate to comorbidity and additional reasons for death among older patients. While older patients were at greater hazard of death compared to younger patients, the SMR declined substantially with age in both men and women. The greater impact of TB on SMRs therefore occurs in the younger population who have few other causes of death. The frequency of other causes of death increased with age among both adults with TB and the general population, and this resulted in the SMR declining with age. Despite a decrease in SMR over time, young women (15–24-year-olds) who had TB, died at a rate 40 times greater than women of the same age without TB in 2016. This SMR was more than double that of men of the same age and is due to differences in both observed and expected mortality. Observed mortality (TB CFR) was 1.5 times greater in women in this age group compared to men. Annually, 250,000 women die during pregnancy in Africa with the majority of deaths due to non-obstetric causes^19^. TB and HIV are among the main infectious causes of maternal deaths^19,20^ and HIV and pregnancy may account for this higher CFR seen in young women. Interpersonal violence among young men in South Africa remains common and among 10–17-year-olds who died due to injury, homicide was the leading cause, mainly affecting young men^21^. This may contribute to the greater than expected mortality among men. In a study from India, the SMR for TB decreased sharply with age from 15.1 among 15–44 year-olds, 7.3 among 45–59-year-olds to 2.2 among those older than 60 years^22^ but was not disaggregated by sex and did not consider HIV^22^. This is consistent with previous work in which the impact of HIV on TB mortality was greatest in younger TB patients^23^. Similarly, a study from Kenya demonstrated that the SMR among TB patients decreased with age but was higher in men than women because of a lower expected mortality among younger men and a higher observed mortality among younger women^24^. The Kenyan study, conducted between 2006 and 2008, reflected a period where women were disproportionately affected by HIV and did not have the benefit of widespread access to ART^24^. In more recent work from Kenya, the SMR among TB patients was again shown to decrease with age; the SMR among older patients (65–74-year-olds) was 7.6 in women and 7.9 in men^25^ comparable with what we observed. The TB SMR in 15–24-year-old women was 25.1 and 14.3 in men of the same age^25^ and reflects a similar difference to what we have shown in this study (Supplementary Table 4).

Regardless of ART use, HIV infection was associated with an increased hazard of TB death. The greater hazard among HIV-positive patients not on ART compared to HIV-positive patients on ART clearly demonstrates the protective effect of ART. Specific concerns regarding men accessing HIV testing, ART services, retention in care, and mortality have previously been raised^6,9^. The higher rate of ART initiation in women compared to men was described from as early as 2004 with the differential between sexes increasing over time^6^. Several systematic reviews have shown that within ART programs (especially in high burden settings), HIV-positive men have a greater hazard of death compared to HIV-positive women^10,11,26^. In South Africa the effect of ART rollout on a rural community has shown more rapid declines in mortality among women than men^9^. The increasing SMR observed in HIV-positive women in this study reflects this greater reduction in general mortality among the population of HIV-positive women who do not have TB. In a South African study reviewing the effect of ART on mortality, SMRs in women were higher than men and this was attributed to the higher background mortality among men^27^. Similarly, we noted much higher SMRs in women, especially in the younger age bands. This reflects the lower number of expected deaths in this patient category (Supplementary Table 5). As South Africa continues to move towards universal access to ART, monitoring of observed and expected mortality should continue across the TB and HIV programs, to track changes over time and identify populations in greatest need of targeted interventions and support.

The importance of adequate history taking, and prioritisation of individuals previously treated for TB is highlighted by the additional risk of death among previously treated TB patients. Extrapulmonary TB was also associated with an increased hazard of death, consistent with earlier data^28,29^ and may be due to more severe and disseminated forms of disease, including TB meningitis.

This study was strengthened by the size of the individual patient data set and the span of 8 years, which provided the opportunity to evaluate temporal changes. We were however careful to only make inferences where biologically plausible, consistent with scientific hypotheses and the literature, and where an effect size was of epidemiological or clinical relevance. We explored effect modification by sex and time in stratified models and while we found differences in point estimates our conclusions were unchanged. Limitations included the use of TB death to refer to death due to any cause during TB treatment, possibly overestimating the effect of TB. However, this is in accordance with international standardised definitions. A large proportion of TB patients had unknown final treatment outcomes, which may have included mortality. Previous work from Malawi has suggested up to 30% of patients lost to follow up may have died^30^. However, by not assuming patients with unknown outcomes had died we have provided conservative CFRs and future work matching individuals with unknown TB treatment outcomes to the population register to ascertain mortality has been planned. A further limitation included the ART information in the TB register. ART use did not differentiate TB patients on ART before TB; those who started ART during TB treatment; or adherence to ART. There was also no information on degree of immunosuppression. We were unable to control for baseline nutritional status, anaemia, underlying disease, or alcohol and drug use as additional risk factors for mortality.

Case fatality during treatment for drug-susceptible TB in South Africa is declining but remains high with 6.9% of TB patients dying during TB treatment in 2016. HIV co-infection, regardless of ART use, increased the hazard of death during TB treatment. We show a disproportionate burden of TB mortality in young women, with a mortality rate among 15–24-year-old women on TB treatment in 2016 more than 40 times greater than for women of the same age in the general population. While future work to prospectively evaluate TB treatment outcomes and risk factors for mortality among young women especially during pregnancy is needed, health services have a clear indication of the need to engage young women during their routine access of health services. Increased education on TB screening, diagnosis, early treatment and treatment completion among young healthy women is needed. Additional care should be offered to young women diagnosed with TB to reduce mortality.

## Supplementary Information

Supplementary Information.
