# Supplementary material for: Mortality during tuberculosis treatment in South Africa using an 8-year analysis of the national tuberculosis treatment register
Source: Sci Rep. 2021 Aug 5;11:15894. doi: 10.1038/s41598-021-95331-w (PMC8342475; doi:10.1038/s41598-021-95331-w)

# Mortality during tuberculosis treatment in South Africa using an eight-year analysis of the national tuberculosis treatment register

Muhammad Osman, Cari van Schalkwyk, Pren Naidoo, James A Seddon, Rory Dunbar, Sicelo S Dlamini, Alex Welte, Anneke C Hesseling, Mareli M Claassens

# Supplementary Material

# Supplementary table 1. Treatment outcomes of adults (≥15 years) treated for drug-susceptible tuberculosis in the South African reporting cohort, 2009-2016

|  | 2009 | 2010 | 2011 | 2012 | 2013 | 2014 | 2015 | 2016 |
| --- | --- | --- | --- | --- | --- | --- | --- | --- |
|  | n (col %) | n (col %) | n (col %) | n (col %) | n (col %) | n (col %) | n (col %) | n (col %) |
|  | 361377 (100) | 360 516 (100) | 353 782 (99.9) | 323 236 (100) | 309 735 (100) | 311 097 (100) | 280 589 (100) | 250 726 (100) |
| Outcome 1^a^ |  | | | | | | | |
| Cured/Completed | 253800 (70.2) | 250 280 (69.4) | 258 068 (72.9) | 240 990 (74.6) | 236 871 (76.5) | 231 044 (74.3) | 224 958 (80.2) | 201 452 (80.3) |
| Died | 37721 (10.4) | 34 990 (9.7) | 33 041 (9.3) | 29 481 (9.1) | 25 013 (8.1) | 22 124 (7.1) | 20 363 (7.3) | 17 314 (6.9) |
| Drug resistance | 1866 (0.5) | 2 007 (0.6) | 2 449 (0.7) | 2 319 (0.7) | 1 930 (0.6) | 2 376 (0.8) | 1 579 (0.6) | 962 (0.4) |
| Failed | 1631 (0.5) | 1 403 (0.4) | 1 391 (0.4) | 1 186 (0.4) | 996 (0.3) | 664 (0.2) | 750 (0.3) | 925 (0.4) |
| Transferred Out | 21582 (6) | 207 60 (5.8) | 20 240 (5.7) | 16 500 (5.1) | 13 786 (4.5) | 11 870 (3.8) | 7 400 (2.6) | 6 438 (2.6) |
| Loss to Follow Up | 44777 (12.4) | 51 076 (14.2) | 38 593 (10.9) | 32 760 (10.1) | 31 139 (10.1) | 43 019 (13.8) | 25 539 (9.1) | 23 635 (9.4) |
| Outcome 2^b^ |  | | | | | | | |
| Favourable | 253800 (70.2) | 250 280 (69.4) | 258 068 (72.9) | 240 990 (74.6) | 236 871 (76.5) | 231 044 (74.3) | 224 958 (80.2) | 201 452 (80.3) |
| Unfavourable | 107577 (29.8) | 110 236 (30.6) | 95 714 (27.1) | 82 246 (25.4) | 72 864 (23.5) | 80 053 (25.7) | 55 631 (19.8) | 49 274 (19.7) |
| Outcome 3^c^ |  | | | | | | | |
| Alive | 257297 (87.2) | 253 690 (87.9) | 261 908 (88.8) | 244 495 (89.2) | 239 797 (90.6) | 234 084 (91.4) | 227 287 (91.8) | 203 339 (92.2) |
| Dead | 37721 (12.8) | 34 990 (12.1) | 33 041 (11.2) | 29 481 (10.8) | 25 013 (9.5) | 22 124 (8.6) | 20 363 (8.2) | 17 314 (7.8) |

1. Outcome 1, as classified in ETR.Net, combining cure and complete for success; and all patients without outcomes as lost to follow up
2. Outcome 2, a binary outcome with success as the only favourable outcome and all other outcomes considered unfavourable
3. Outcome 3, patients in whom a final vitality status is unknown (lost to follow up, moved or not evaluated) n = 409,114 were excluded

# Supplementary table 2. Multivariable Cox regression model predicting adjusted hazard ratio of death among adults (≥15 years) with drug-susceptible TB and known vitality status stratified by sex, South Africa, 2009-2016 (n = 2,122,419)

|  | | aHR (95%CI) | |
| --- | --- | --- | --- |
|  |  | Males  n = 1,172,409 | Females  n = 950,010 |
| Age category | 15-24 years | reference | |
|  | 25-34 years | 1.7 (1.65-1.76) | 1.3 (1.27-1.33) |
|  | 35-44 years | 2.04 (1.98-2.11) | 1.49 (1.45-1.52) |
|  | 45-54 years | 2.65 (2.57-2.74) | 1.83 (1.78-1.87) |
|  | 55-64 years | 4.05 (3.91-4.18) | 2.6 (2.53-2.68) |
|  | 65+ years | 7.04 (6.8-7.29) | 4.1 (3.97-4.22) |
| HIV status | HIV-negative | reference | |
|  | HIV unknown | 2 (1.96-2.04) | 2.47 (2.41-2.53) |
|  | HIV-positive not on ART | 2.58 (2.53-2.63) | 2.79 (2.72-2.85) |
|  | HIV-positive on ART | 1.76 (1.73-1.79) | 2 (1.95-2.04) |
| Previous TB treatment | New | reference | |
|  | Retreatment | 1.26 (1.24-1.28) | 1.28 (1.26-1.3) |
| Site of disease | PTB | reference | |
|  | EPTB | 1.41 (1.39-1.43) | 1.4 (1.38-1.42) |
| Year of TB treatment | 2009 | reference | |
|  | 2010 | 0.97 (0.95-0.99) | 0.98 (0.95-1) |
|  | 2011 | 0.96 (0.94-0.98) | 0.91 (0.89-0.93) |
|  | 2012 | 0.97 (0.95-0.99) | 0.95 (0.92-0.97) |
|  | 2013 | 0.92 (0.9-0.95) | 0.93 (0.91-0.96) |
|  | 2014 | 0.9 (0.88-0.92) | 0.89 (0.86-0.91) |
|  | 2015 | 0.85 (0.83-0.87) | 0.87 (0.84-0.89) |
|  | 2016 | 0.8 (0.78-0.82) | 0.84 (0.81-0.87) |

p-values not shown, all p-values in multi variable model significant <0.05

ART: antiretroviral therapy; EPTB: extrapulmonary TB; PTB: pulmonary TB; TB: tuberculosis

# Supplementary Table 3. Multivariable Cox regression model predicting adjusted hazard ratio of death among adults (≥15 years) with drug-susceptible TB and known vitality status stratified by year, South Africa, 2009-2016 (n = 2,122,419)

|  | | aHR (95%CI) | | | | | | | | |
| --- | --- | --- | --- | --- | --- | --- | --- | --- | --- | --- |
|  |  | 2009  n = 291,600 | 2010  n = 285,348 | 2011  n = 292,643 | 2012  n = 271,845 | 2013  n = 262,554 | 2014  n = 251,812 | 2015  n = 246,557 | 2016  n = 220,060 |  |
| Age category | 15-24 years | reference | | | | | | | |  |
|  | 25-34 years | 1.56 (1.5-1.63) | 1.38 (1.32-1.44) | 1.32 (1.26-1.39) | 1.45 (1.37-1.52) | 1.42 (1.34-1.5) | 1.38 (1.3-1.47) | 1.4 (1.31-1.5) | 1.38 (1.28-1.48) |  |
|  | 35-44 years | 1.75 (1.67-1.83) | 1.52 (1.45-1.59) | 1.54 (1.47-1.62) | 1.69 (1.6-1.78) | 1.72 (1.62-1.82) | 1.63 (1.53-1.73) | 1.78 (1.67-1.9) | 1.71 (1.59-1.83) |  |
|  | 45-54 years | 2.04 (1.95-2.14) | 1.88 (1.8-1.97) | 1.96 (1.87-2.06) | 2.16 (2.04-2.28) | 2.3 (2.16-2.44) | 2.23 (2.1-2.38) | 2.38 (2.23-2.54) | 2.26 (2.1-2.43) |  |
|  | 55-64 years | 2.73 (2.6-2.88) | 2.73 (2.59-2.87) | 2.84 (2.69-3) | 3.41 (3.22-3.61) | 3.55 (3.33-3.78) | 3.4 (3.18-3.63) | 3.68 (3.44-3.95) | 3.64 (3.38-3.93) |  |
|  | 65+ years | 3.93 (3.72-4.15) | 4.15 (3.92-4.38) | 4.81 (4.54-5.09) | 5.96 (5.61-6.34) | 6.06 (5.67-6.47) | 6.07 (5.67-6.5) | 6.44 (5.99-6.92) | 6.42 (5.94-6.94) |  |
| Sex | Male | reference | | | | | | | |  |
|  | Female | 0.98 (0.96-1) | 0.96 (0.94-0.98) | 0.89 (0.87-0.91) | 0.92 (0.9-0.94) | 0.95 (0.93-0.98) | 0.92 (0.9-0.95) | 0.96 (0.94-0.99) | 0.98 (0.95-1.01) |  |
| HIV status | HIV-negative | reference | | | | | | | |  |
|  | HIV unknown | 2.24 (2.16-2.33) | 2.28 (2.19-2.36) | 2.14 (2.06-2.22) | 1.96 (1.88-2.05) | 2.01 (1.91-2.12) | 2.27 (2.14-2.41) | 2.28 (2.13-2.45) | 1.81 (1.67-1.97) |  |
|  | HIV-positive not on ART | 2.6 (2.5-2.71) | 2.56 (2.47-2.66) | 2.42 (2.34-2.51) | 2.51 (2.42-2.6) | 2.89 (2.78-3.02) | 2.8 (2.68-2.93) | 5.11 (4.84-5.4) | 6.12 (5.75-6.5) |  |
|  | HIV-positive on ART | 1.94 (1.85-2.04) | 1.88 (1.81-1.96) | 2.02 (1.95-2.1) | 1.98 (1.92-2.05) | 1.88 (1.82-1.95) | 1.82 (1.76-1.88) | 1.85 (1.79-1.92) | 1.87 (1.8-1.95) |  |
| Previous TB treatment | New | reference | | | | | | | |  |
|  | Retreatment | 1.33 (1.3-1.37) | 1.33 (1.3-1.37) | 1.25 (1.22-1.28) | 1.34 (1.3-1.38) | 1.28 (1.23-1.33) | 1.09 (1.04-1.15) | 1.09 (1.04-1.14) | 1.09 (1.04-1.15) |  |
| Site of disease | PTB | reference | | | | | | | |  |
|  | EPTB | 1.36 (1.32-1.39) | 1.41 (1.37-1.45) | 1.37 (1.33-1.41) | 1.42 (1.37-1.46) | 1.48 (1.43-1.53) | 1.49 (1.44-1.54) | 1.39 (1.34-1.45) | 1.37 (1.32-1.43) |  |

p-values not shown, all p-values in multi variable model significant <0.05 except for females in 2016 p = 0.22

ART: antiretroviral therapy; EPTB: extrapulmonary TB; PTB: pulmonary TB; TB: tuberculosis

# Supplementary table 4. Tuberculosis cases, observed deaths and expected deaths of adults (≥15 years) treated for drug-susceptible tuberculosis in the South African reporting cohort by age and sex, 2009-2016

| Year | Age category | TB Cases | | Observed TB deaths | | Expected deaths^a^ | | TB mortality rate^b^ | | Population mortality rate^b^ | | Standardised mortality ratio^c^ | |
| --- | --- | --- | --- | --- | --- | --- | --- | --- | --- | --- | --- | --- | --- |
|  |  | Female | Male | Female | Male | Female | Male | Female | Male | Female | Male | Female | Male |
| 2009 | 15-24 years | 30582 | 20124 | 1919 | 793 | 34 | 27.1 | 13.83 | 8.67 | 0.25 | 0.30 | 56.4 | 29.3 |
| 2010 | 15-24 years | 30229 | 19901 | 1851 | 750 | 30.8 | 24.3 | 13.88 | 8.48 | 0.23 | 0.28 | 60.1 | 30.8 |
| 2011 | 15-24 years | 28350 | 19270 | 1562 | 678 | 28.1 | 23.4 | 11.92 | 7.64 | 0.21 | 0.26 | 55.7 | 29 |
| 2012 | 15-24 years | 25544 | 18215 | 1230 | 574 | 24.2 | 22.2 | 10.27 | 6.74 | 0.20 | 0.26 | 50.7 | 25.9 |
| 2013 | 15-24 years | 23268 | 17873 | 987 | 506 | 20.6 | 21.2 | 9.20 | 6.18 | 0.19 | 0.26 | 47.8 | 23.9 |
| 2014 | 15-24 years | 22632 | 18279 | 851 | 489 | 17.7 | 19.8 | 8.92 | 6.35 | 0.19 | 0.26 | 48.1 | 24.6 |
| 2015 | 15-24 years | 20107 | 16804 | 731 | 437 | 17.1 | 19.8 | 7.81 | 5.65 | 0.18 | 0.26 | 42.8 | 22 |
| 2016 | 15-24 years | 17126 | 15436 | 597 | 377 | 14.1 | 18.1 | 7.55 | 5.31 | 0.18 | 0.26 | 42.3 | 20.8 |
| 2009 | 25-34 years | 60410 | 56189 | 6019 | 4982 | 273.4 | 247.8 | 22.03 | 19.99 | 1.00 | 0.99 | 22 | 20.1 |
| 2010 | 25-34 years | 59702 | 55487 | 5345 | 4509 | 230.1 | 213.9 | 20.09 | 18.55 | 0.87 | 0.88 | 23.2 | 21.1 |
| 2011 | 25-34 years | 56648 | 53886 | 4607 | 4007 | 193.7 | 196.6 | 17.29 | 16.13 | 0.73 | 0.79 | 23.8 | 20.4 |
| 2012 | 25-34 years | 50180 | 50957 | 3932 | 3680 | 148.8 | 176.2 | 16.59 | 15.54 | 0.63 | 0.74 | 26.4 | 20.9 |
| 2013 | 25-34 years | 45519 | 49659 | 3117 | 2973 | 113.4 | 159.6 | 14.89 | 13.15 | 0.54 | 0.71 | 27.5 | 18.6 |
| 2014 | 25-34 years | 43917 | 50850 | 2533 | 2676 | 89.3 | 142 | 13.69 | 12.75 | 0.48 | 0.68 | 28.4 | 18.8 |
| 2015 | 25-34 years | 37630 | 45696 | 2231 | 2286 | 81 | 137.4 | 12.78 | 10.95 | 0.46 | 0.66 | 27.6 | 16.6 |
| 2016 | 25-34 years | 32532 | 42123 | 1820 | 2006 | 67.8 | 122.8 | 12.07 | 10.42 | 0.45 | 0.64 | 26.8 | 16.3 |
| 2009 | 35-44 years | 40829 | 58909 | 4556 | 6307 | 239.6 | 463.2 | 24.29 | 23.71 | 1.28 | 1.74 | 19 | 13.6 |
| 2010 | 35-44 years | 40977 | 58560 | 4082 | 5644 | 211.1 | 402.8 | 22.01 | 21.70 | 1.14 | 1.55 | 19.3 | 14 |
| 2011 | 35-44 years | 40474 | 57491 | 3770 | 5540 | 189.8 | 369.6 | 19.46 | 20.67 | 0.98 | 1.38 | 19.9 | 15 |
| 2012 | 35-44 years | 35323 | 53549 | 3290 | 4893 | 145.4 | 323.3 | 19.46 | 19.43 | 0.86 | 1.28 | 22.6 | 15.1 |
| 2013 | 35-44 years | 32743 | 52640 | 2695 | 4175 | 114.3 | 287.9 | 17.69 | 17.45 | 0.75 | 1.20 | 23.6 | 14.5 |
| 2014 | 35-44 years | 31948 | 52920 | 2268 | 3524 | 90.9 | 253.4 | 16.75 | 15.87 | 0.67 | 1.14 | 24.9 | 13.9 |
| 2015 | 35-44 years | 27920 | 48192 | 2125 | 3353 | 83.7 | 243.9 | 16.31 | 15.16 | 0.64 | 1.10 | 25.4 | 13.7 |
| 2016 | 35-44 years | 24005 | 43721 | 1765 | 2799 | 70.2 | 214.4 | 15.69 | 13.88 | 0.62 | 1.06 | 25.2 | 13.1 |
| 2009 | 45-54 years | 20794 | 36623 | 2502 | 4489 | 133.1 | 348.5 | 26.08 | 26.99 | 1.39 | 2.10 | 18.8 | 12.9 |
| 2010 | 45-54 years | 21175 | 36598 | 2342 | 4246 | 125.2 | 318.9 | 24.41 | 26.01 | 1.31 | 1.95 | 18.7 | 13.3 |
| 2011 | 45-54 years | 21181 | 36479 | 2247 | 4169 | 120 | 307.1 | 22.40 | 24.75 | 1.20 | 1.82 | 18.7 | 13.6 |
| 2012 | 45-54 years | 18593 | 33530 | 1989 | 3658 | 97.9 | 273.6 | 22.50 | 23.34 | 1.11 | 1.75 | 20.3 | 13.4 |
| 2013 | 45-54 years | 17843 | 32618 | 1783 | 3169 | 82.8 | 249.2 | 21.93 | 21.34 | 1.02 | 1.68 | 21.5 | 12.7 |
| 2014 | 45-54 years | 17963 | 33448 | 1511 | 2968 | 72.7 | 227.6 | 19.74 | 21.17 | 0.95 | 1.62 | 20.8 | 13 |
| 2015 | 45-54 years | 15996 | 31012 | 1405 | 2715 | 68 | 224.8 | 18.97 | 19.18 | 0.92 | 1.59 | 20.7 | 12.1 |
| 2016 | 45-54 years | 13958 | 27951 | 1199 | 2268 | 58 | 200.1 | 18.52 | 17.61 | 0.90 | 1.55 | 20.7 | 11.3 |
| 2009 | 55-64 years | 8925 | 15291 | 1305 | 2360 | 70.8 | 188.9 | 32.48 | 34.99 | 1.76 | 2.80 | 18.4 | 12.5 |
| 2010 | 55-64 years | 9283 | 15647 | 1299 | 2386 | 69.8 | 185.2 | 32.04 | 35.30 | 1.72 | 2.74 | 18.6 | 12.9 |
| 2011 | 55-64 years | 9758 | 16363 | 1263 | 2480 | 73.3 | 196.5 | 28.41 | 33.61 | 1.65 | 2.66 | 17.2 | 12.6 |
| 2012 | 55-64 years | 8638 | 15506 | 1169 | 2359 | 63.2 | 181.9 | 29.64 | 33.95 | 1.60 | 2.62 | 18.5 | 13 |
| 2013 | 55-64 years | 8627 | 15614 | 1036 | 2165 | 59.8 | 177.2 | 26.85 | 31.46 | 1.55 | 2.58 | 17.3 | 12.2 |
| 2014 | 55-64 years | 8681 | 16378 | 959 | 1974 | 54.2 | 169 | 26.65 | 29.64 | 1.51 | 2.54 | 17.7 | 11.7 |
| 2015 | 55-64 years | 8131 | 15682 | 927 | 1901 | 54.4 | 174.4 | 25.24 | 27.35 | 1.48 | 2.51 | 17.1 | 10.9 |
| 2016 | 55-64 years | 7194 | 14415 | 781 | 1696 | 47.7 | 160 | 23.89 | 26.30 | 1.46 | 2.48 | 16.4 | 10.6 |
| 2009 | 65+ years | 5775 | 6926 | 951 | 1538 | 125.6 | 180.1 | 38.16 | 54.24 | 5.04 | 6.35 | 7.6 | 8.5 |
| 2010 | 65+ years | 6003 | 6954 | 1025 | 1511 | 126.1 | 178.6 | 40.75 | 53.61 | 5.02 | 6.34 | 8.1 | 8.5 |
| 2011 | 65+ years | 6543 | 7339 | 1090 | 1628 | 139.6 | 189.4 | 38.49 | 53.49 | 4.93 | 6.22 | 7.8 | 8.6 |
| 2012 | 65+ years | 6151 | 7050 | 1105 | 1602 | 130.1 | 180.7 | 41.82 | 54.97 | 4.93 | 6.20 | 8.5 | 8.9 |
| 2013 | 65+ years | 6161 | 7170 | 1015 | 1392 | 127.7 | 181.5 | 39.05 | 47.35 | 4.91 | 6.17 | 8 | 7.7 |
| 2014 | 65+ years | 6348 | 7733 | 903 | 1468 | 122.6 | 178.2 | 36.06 | 50.59 | 4.89 | 6.14 | 7.4 | 8.2 |
| 2015 | 65+ years | 6103 | 7316 | 884 | 1368 | 130.1 | 187.9 | 33.14 | 44.50 | 4.88 | 6.11 | 6.8 | 7.3 |
| 2016 | 65+ years | 5438 | 6827 | 779 | 1227 | 116 | 177.9 | 32.60 | 41.93 | 4.85 | 6.08 | 6.7 | 6.9 |

SMR: Standardised mortality ratio; TB: tuberculosis

1. Expected deaths are the product of Thembisa age and sex specific estimates of mortality rates for the general population and person time recorded in the TB cohort
2. Mortality rates expressed per 100 person years
3. Standardised mortality ratio is the ratio of observed TB deaths to the expected deaths in the TB cohort based on the Thembisa estimates of mortality rates for the general population

# Supplementary table 5. Tuberculosis cases, observed deaths and expected deaths of adults (≥15 years) treated for drug-susceptible tuberculosis in the South African reporting cohort by HIV and sex, 2009-2016

| Year | HIV status | TB Cases | | Observed TB deaths | | Expected deaths^a^ | | TB mortality rate^b^ | | Population mortality rate^b^ | | Standardised mortality ratio^c^ | |
| --- | --- | --- | --- | --- | --- | --- | --- | --- | --- | --- | --- | --- | --- |
|  |  | Female | Male | Female | Male | Female | Male | Female | Male | Female | Male | Female | Male |
| 2009 | HIV- | 22607 | 36400 | 1104 | 2110 | 114.2 | 215 | 10.25 | 12.25 | 1.06 | 1.25 | 9.67 | 9.81 |
| 2010 | HIV- | 29955 | 48134 | 1453 | 2812 | 146.6 | 271.1 | 10.44 | 12.69 | 1.05 | 1.22 | 9.91 | 10.37 |
| 2011 | HIV- | 35199 | 57896 | 1765 | 3561 | 174.8 | 327.9 | 10.49 | 13.02 | 1.04 | 1.2 | 10.1 | 10.86 |
| 2012 | HIV- | 34212 | 58457 | 1857 | 3664 | 171.8 | 332.9 | 11.31 | 13.24 | 1.05 | 1.2 | 10.81 | 11.01 |
| 2013 | HIV- | 35342 | 62708 | 1829 | 3593 | 173.2 | 347.3 | 11.13 | 12.5 | 1.05 | 1.21 | 10.56 | 10.35 |
| 2014 | HIV- | 36531 | 67285 | 1739 | 3657 | 167.7 | 346.2 | 11.02 | 12.83 | 1.06 | 1.21 | 10.37 | 10.57 |
| 2015 | HIV- | 33881 | 64237 | 1599 | 3437 | 169.4 | 357.5 | 10.11 | 11.73 | 1.07 | 1.22 | 9.44 | 9.61 |
| 2016 | HIV- | 29891 | 59280 | 1351 | 3046 | 150.4 | 332.1 | 9.7 | 11.25 | 1.08 | 1.23 | 8.99 | 9.17 |
| 2009 | HIV+ | 73751 | 66714 | 8243 | 8284 | 917.4 | 1176 | 24.63 | 27.75 | 2.74 | 3.94 | 8.99 | 7.04 |
| 2010 | HIV+ | 98424 | 90416 | 10119 | 10302 | 1023 | 1316.2 | 23 | 25.92 | 2.33 | 3.31 | 9.89 | 7.83 |
| 2011 | HIV+ | 107279 | 102206 | 10489 | 11686 | 948.9 | 1301.9 | 20.78 | 24.81 | 1.88 | 2.76 | 11.05 | 8.98 |
| 2012 | HIV+ | 97508 | 99214 | 9545 | 10945 | 705.7 | 1113.1 | 20.78 | 23.8 | 1.54 | 2.42 | 13.53 | 9.83 |
| 2013 | HIV+ | 90383 | 98433 | 7920 | 9482 | 506.5 | 948.5 | 19.06 | 21.25 | 1.22 | 2.13 | 15.64 | 10 |
| 2014 | HIV+ | 89668 | 103050 | 6736 | 8615 | 376.7 | 813.9 | 17.86 | 20.2 | 1 | 1.91 | 17.88 | 10.59 |
| 2015 | HIV+ | 78703 | 94355 | 6361 | 8008 | 336 | 767.6 | 17.46 | 18.57 | 0.92 | 1.78 | 18.93 | 10.43 |
| 2016 | HIV+ | 67623 | 85830 | 5345 | 6912 | 272.9 | 652.3 | 17.08 | 17.54 | 0.87 | 1.66 | 19.59 | 10.6 |

SMR: Standardised mortality ratio; TB: tuberculosis

1. Expected deaths are the product of Thembisa HIV and sex specific estimates of mortality rates for the general population and person time recorded in the TB cohort
2. Mortality rates expressed per 100 person years
3. Standardised mortality ratio is the ratio of observed TB deaths to the expected deaths in the TB cohort based on the Thembisa estimates of mortality rates for the general population.

# Supplementary figure 1. Changes in HIV testing and antiretroviral therapy use among adults (≥15 years) on drug-susceptible tuberculosis treatment between 2009 and 2016 in South Africa


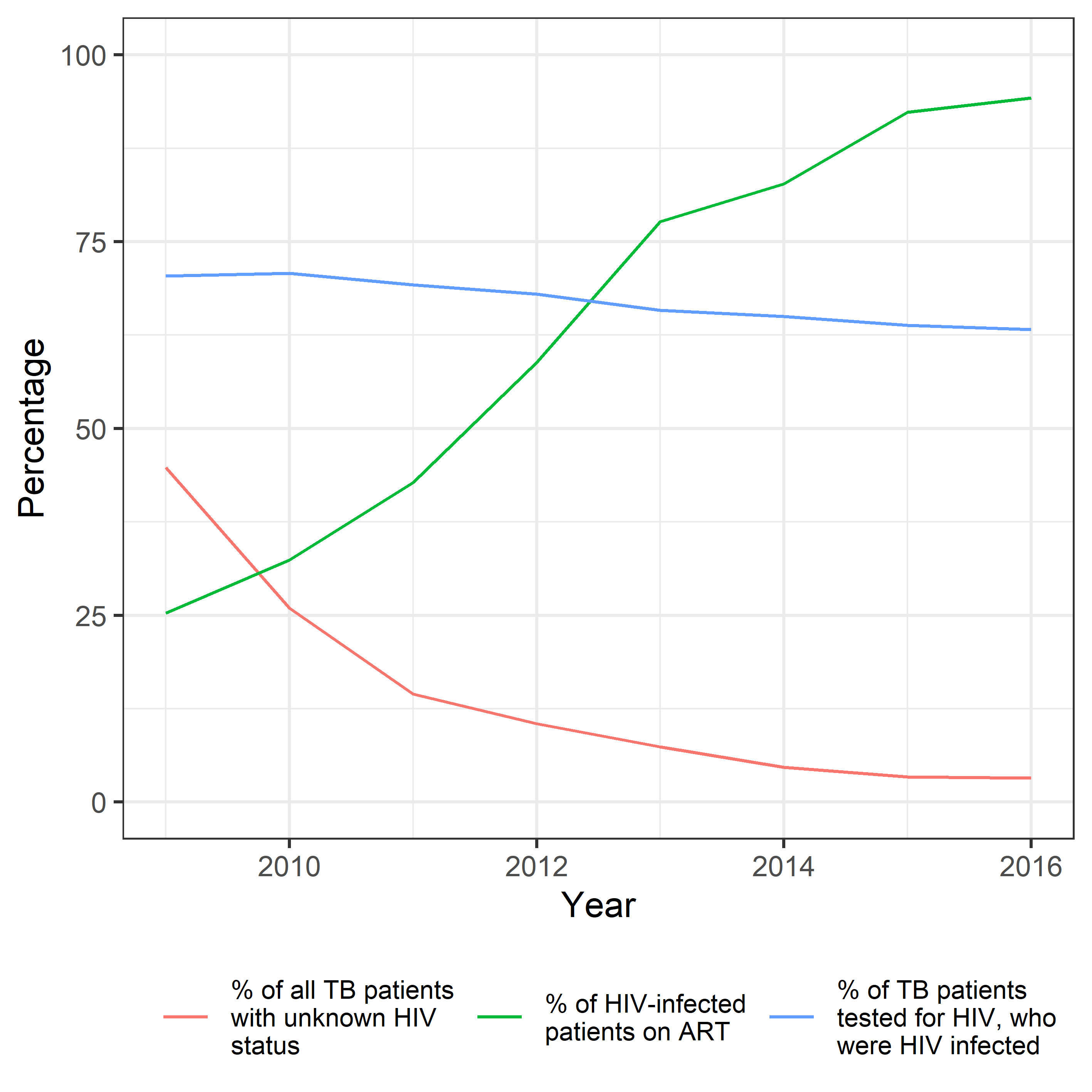


# Supplementary figure 2. Standardised mortality ratios by age and sex of adults (≥15 years) on drug-susceptible tuberculosis treatment in South Africa, 2009-2016


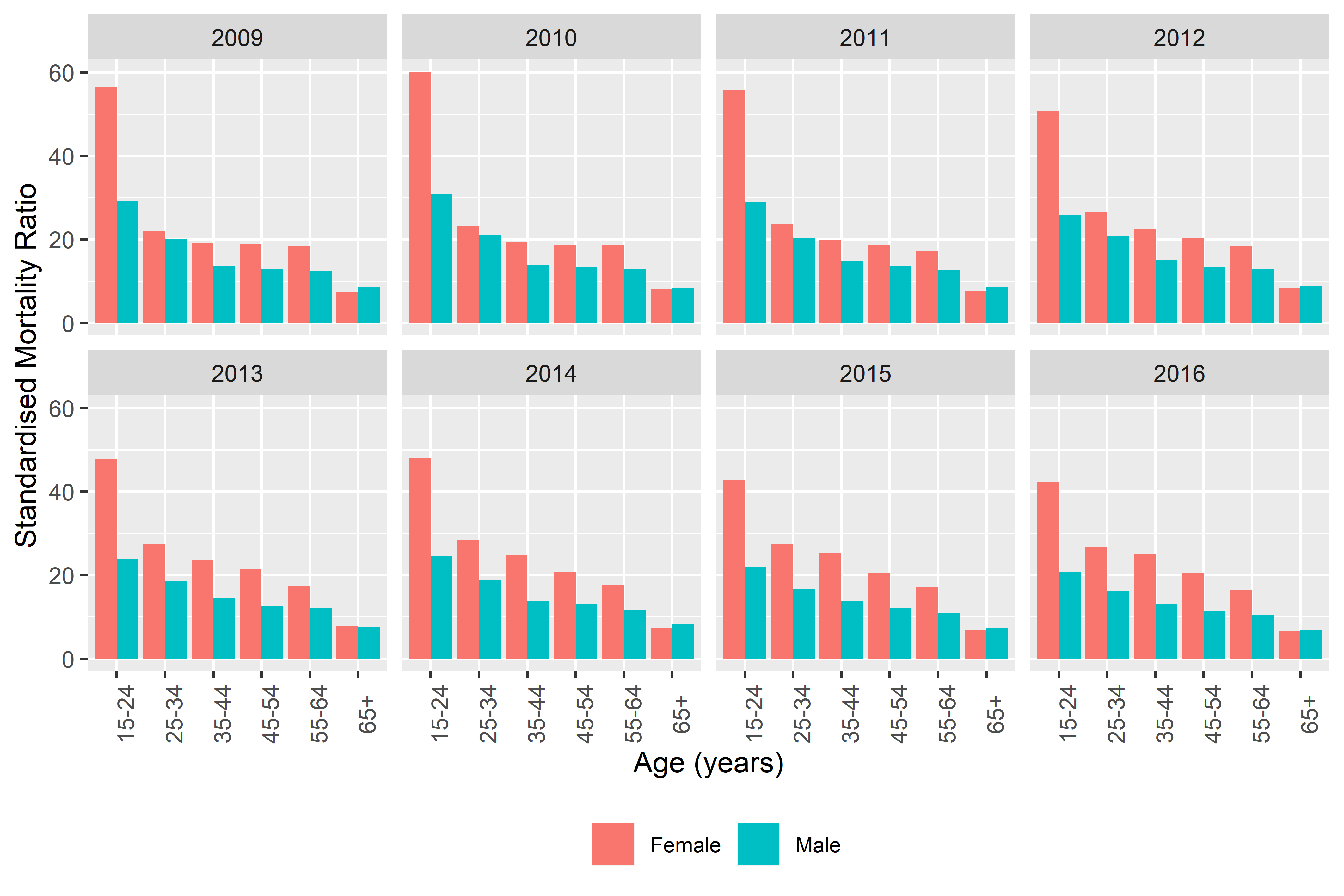

Supplement: Supplementary file 1 — Supplementary Information. [file 41598_2021_95331_MOESM1_ESM.docx]
